# Successful implementation of infection control measure in a neonatal intensive care unit to combat the spread of pathogenic multidrug resistant *Staphylococcus capitis*

**DOI:** 10.1186/s13756-019-0512-8

**Published:** 2019-03-27

**Authors:** Jérôme Ory, Michel Cazaban, Brigitte Richaud-Morel, Massimo Di Maio, Catherine Dunyach-Remy, Alix Pantel, Albert Sotto, Frédéric Laurent, Jean-Philippe Lavigne, Marine Butin

**Affiliations:** 1 Department of Infection Control, Equipe Opérationnelle d’Hygiène, Place du Professeur Robert Debré, University Hospital Nîmes, Nîmes, France; 2 Department of Neonatalogy, University Hospital Nîmes, Nîmes, France; 3 Bacterial virulence and Infectious Diseases, INSERM, Department of Microbiology, Université de Montpellier, University Hospital Nîmes, Nîmes cedex 02, 30908 Nîmes, France; 4 Department of Infectious Diseases, Bacterial virulence and Infectious Diseases, INSERM, Université de Montpellier, University Hospital Nîmes, Nîmes, France; 5 International Center of Research in Infectiology, INSERM U1111, CNRS UMR5308, Ecole Normale Supérieure, Lyon, France; 6 Institute of Infectious Agents, Lyon, France; 7 National Reference Center for Staphylococci, Lyon, France; 8 Department of Microbiology-Mycology, Institut des Sciences Pharmaceutiques et Biologiques de Lyon, University of Lyon, Lyon, France

**Keywords:** *Staphylococcus capitis*, Neonatal intensive care unit, Steam cleaner, Healthcare-associated infection, Disinfection

## Abstract

**Background:**

Once present in a neonatal intensive care unit (NICU), multidrug resistant *Staphylococcus capitis* NRCS-A is able to settle and diffuse.

**Objective:**

The objective of this study was to evaluate the impact of infection control (IC) interventions to reduce the spread of *Staphylococcus capitis* NRCS-A in a NICU.

**Methods:**

Between December 2012 and December 2017, all patients presenting positive sampling (blood, skin or catheter) to *S. capitis* were included, and clinical data were recorded from electronic clinical charts. The IC team has continually implemented measures of control infections (hand hygiene, standard precautions, patient contact isolation and disinfection of the inanimate environment). From May 2015, a steam cleaner was implemented in the cleaning procedure instead of disinfectant to disinfect heating tables and incubators. Four periods were determined: Period 1 (P1) before steam cleaner acquisition; Period 2 (P2) after implementation steam cleaner; Period 3 (P3) when the steam cleaner had broken down, and Period 4 (P4) when the steam cleaner was functional again. The consumption of antibiotics and the epidemiology of infections inside the NICU were investigated during the study period.

**Results:**

During the studied period, 37 infants were infected or colonized by *S. capitis*. The incidences of infection or colonization by *S. capitis* were P1 = 1.04‰, P2 = 0.55‰, P3 = 3.95 ‰ and P4 = 0‰ and were significantly different between P1-P3 and P2-P4 (*p* < 0.001). During the different periods, antibiotics consumption and bacterial epidemiology of the ward were stable.

**Conclusions:**

The use of steam vapor system was associated with a significantly decreased incidence of *S. capitis* NRCS-A infection or colonization and could constitute an effective and safe procedure to control and eradicate its diffusion inside NICUs.

## Background

Healthcare-Acquired Infections (HAIs) represent a frequent issue in infants hospitalized in Neonatal Intensive Care Units (NICUs) with rates from 8 to 30% [1] and are often associated with substantial morbidity and mortality. Associated risk factors include low birth weight, indwelling central catheter, parenteral nutrition, prior antibiotic exposure, and invasive procedures [2]. In NICUs, the predominant organisms involved in HAIs are coagulase-negative staphylococci (CoNS) [3]. Among them, *Staphylococcus capitis* clone NRCS-A has been previously described as an emerging cause of HAIs and has been isolated worldwide, specifically in NICU [4–6]. In our university hospital, sporadic cases as well as series of neonatal sepsis involving *S. capitis* clone NRCS-A have been reported since 2012.

These bacteria may constitute a major challenge due to high levels of antimicrobial resistance raising therapeutic concerns in case of late-onset sepsis [6]. All *S. capitis* NRCS-A strains are resistant to beta-lactams and exhibit reduced susceptibility to other antimicrobial agents commonly used in NICUs, including resistance or heteroresistance to vancomycin [6].

A large proportion of HAIs are preventable by the implementation of multimodal infection prevention and control strategies. These strategies include hand hygiene, standard precautions and environmental cleaning [7]. One of the most common source of transmission and contamination is represented by environmental surfaces. Such environmental colonization may play a significant role in the transmission of *S. capitis* NRCS-A between hospitalized infants and its persistence after introduction in a NICU. A previous study revealed that some *Staphylococcus* strains such as *S. capitis* survived very well on inert surfaces [8]. The high-level of resistance of this clone to detergent molecules could also be involved in its persistence in hospital environments and in the recurrence of local outbreaks in some NICUs [9].

The usual prevention methods and hygiene procedures have not allowed to control the diffusion of the clone NRCS-A inside NICUs. Thus, the cleaning procedure to disinfect heating tables and incubators was changed from classical procedure based on detergent molecules to the use of a steam cleaner from April 2015. A study has demonstrated the efficiency of this steam cleaner [10].

The objective of this study was to evaluate the impact of infection control (IC) interventions to reduce the spread of *S. capitis* NRCS-A during a 5-years period in our NICU.

## Methods

### Study design and eligibility criteria

This retrospective study was performed in the two units (special care nurseries (15 beds) and classical intensive care (15 beds)) of NICUs in the French University Hospital of Nîmes, from 1st December 2012 to 31st December 2017. A total of 2416 patients admitted in NICUs were hospitalized in these settings during the study.

### Study population-total neonates admitted

For all patients presenting positive sampling (blood, skin or catheter cultures) to *S. capitis*, clinical data were collected (sex, gestational age, head circumference, weight, intrauterine growth restriction, prophylactic antibiotic during the NICU stay, antibiotherapy and death considered to be related to *S. capitis* infection) from electronic clinical charts. The patients were then classified as “infected” or “colonized” with *S. capitis* by an independent neonatologist and IC practitioner on the basis of the clinical and biological context (symptoms, CRP level, antibiotic administration and duration, follow-up of the patient).

### Infection control interventions

Because of the observation of several *S. capitis* sepsis cases in the NICU since 2012, the IC department has continually implemented various measures of prevention and control of infections including intensified hand hygiene, patient contact isolation and disinfection of the inanimate environment with a sodium hypochlorite solution. Moreover, trainings on standard precautions were performed for healthcare workers (paediatric nurses, nurses and housekeepers).

From May 2015, a steam cleaner (Sanivap SV2900) was used in the cleaning procedure to disinfect heating tables and incubators, instead of the classical procedure based on detergent molecules. Four periods were thus determined: i) Period 1 (P1): period before steam cleaner acquisition (December 2012–April 2015), ii) Period 2 (P2): period of use of steam cleaner (May 2015–November 2016), iii) Period 3 (P3): period when steam cleaner was out of order (December 2016–February 2017), iv) Period 4 (P4) corresponding to the period when the steam cleaner was functional again (March 2017–December 2017). The incidence of *S. capitis* infection or colonisation (ratio between number of patients infected or colonized and number of total person-hospital days) was calculated for each period. Furthermore, the consumptions of vancomycin and linezolid were surveyed during this study by CONSORES software (www.consores.net).

### Microbial analysis (identification, antimicrobial susceptibility profile and typing)

*S. capitis* isolates were identified by matrix-assisted laser desorption/ionization time-of-flight mass spectrometry (MALDI-TOF MS, Vitek MS, bioMerieux, France). Antibiotic susceptibility tests were performed using the disk diffusion method (BioRad, France) on Mueller-Hinton agar according to recommendations of EUCAST-CASFM (European Committee on Antimicrobial Susceptibility Testing (EUCAST), Comité de l’Antibiogramme de la Société Francaise de Microbiologie (SFM) (http://www.sfm-microbiologie.org). Minimum Inhibitory Concentrations (MICs) were determined using the E-test method (bioMérieux) for linezolid and UMIC (Unitary Minimum Inhibitory Concentrations) method (Biocentric, France) for teicoplanin and vancomycin. Methicillin resistance was identified using cefoxitin disks (30 μg). Molecular typing of the isolates was performed using Pulse Field Gel Electrophoresis (PFGE) analysis as previously published [11]. The bacterial epidemiology of all infections (positive blood cultures) observed during the study period was recorded from the laboratory data.

### Statistical analysis

Statistical analysis of incidences of *S. capitis* infection or colonisation was carried out to compare the impact of infection control (IC) interventions to reduce the spread of *S. capitis* NRCS-A according to period. The means of the other microorganisms (CoNS and *S. aureus*) were also compared between periods. Comparisons between incidences were conducted using Fisher’s exact test. A two-sided *P*-value < 0.001 was considered to indicate significance. Statistical analyses were performed using R studio software (version 3.0.2).

## Results

### Characteristics of population-total neonates admitted in the study

A total of 2518 patients were admitted in the NICU setting among which 37 infants (mean gestational age 27 weeks) were infected or colonized with *S. capitis.* The first case was noted in December 2012. Among these 37 infants, 6 (16.2%) died, including 2 deaths considered to be related to *S. capitis* infections. The demographic data are presented in Table 1.

**Table 1:** Epidemiological, clinical and biological characteristics of preterm infants infected/colonized by *Staphylococcus capitis* NRCS-A clone in Neonatal Intensive Care Unit (NICU) at Nîmes University Hospital from 2012 to 2017

Characteristics	Period 1 No steam cleaner	Period 2 Steam cleaner	Period 3 No steam cleaner	Period 4 Steam cleaner	Total
	Dec 12-Apr 15	May 15-Nov 16	Dec 16-Feb 17	Mar 17-Dec 17	Dec 12-Dec 17
Number of patients (*n*)	23	7	7	0	37
Number of patients admitted to the NICU	1255	747	100	416	2518
Incidence of *S. capitis* (‰)	1.04	0.55	3.95	0*	0.85
Male, *n* (%)	14 (60.9)	5 (71.4)	5 (71.4)	–	24 (64.9)
Gestational age, weeks (range)	27 (25–35)	28 (24–33)	27 (24–29)	–	27 (25–34)
Body weight, g (range)	919 (560–2500)	1175 (790–1985)	1095 (680–1400)	–	995(560–2500)
Intrauterine growth restriction, *n* (%)	7 (30.4)	1 (14.3)	0 (0)	–	8 (21.6)
Death, *n* (%)	4 (17.4)	0 (0)	2 (28.6)	0 (0)	6 (16.2)
Death due to *S. capitis*, *n* (%)	2 (8.7)	0 (0)	0 (0)	0 (0)	2 (5.4)
Infection *S. aureus*	1.7	1.6	1.7	1.7	1.7
Infection *S. capitis*, *n* (%)	9 (39.1)	1 (14.3)	2 (28.6)	–	12 (32.4)
Main sites of isolation					
Blood, *n* (%)	7 (78)	1 (100)	2 (100)	–	10 (83)
Catheter, *n* (%)	2 (22)	0 (0)	0 (0)		2 (17)
Cutaneous, *n* (%)	0 (0)	0 (0)	0 (0)	–	0
Colonisation *S. capitis*	14	6	5	–	
Main sites of isolation					
Blood, *n* (%)	3 (26)	0 (0)	0 (0)	–	3 (12)
Catheter, *n* (%)	9 (60)	6 (100)	5 (100)	–	20 (80)
Cutaneous, *n* (%)	2 (14)	0 (0)	0 (0)	–	2 (8)
Number of patients which have received antibiotics treatment before infection, *n* (%)	16 (69.6)	4 (57.1)	5 (71.4)	–	25 (67.6)
Number of patients which have received vancomycin used before infection, *n* (%)	8 (34.8)	2 (28.6)	3 (42.9)	–	13 (35.1)
DDD/1000HD^a^ Vancomycin	25.4	25.5	29.1	27.0	27.4
DDD/1000HD Linezolid	12.1	9.3	14.0	11.7	11.4

*p < 0.001, comparison between incidences during the different periods (Period 1, Period 2, Period 3 and Period 4) using Fisher’s exact test; ^a^DDD/1000HD: Defined Daily Dose/1000 Hospital Days

### Results as regards infection or colonization

From December 1st 2012 to December 31st 2017, four periods were individualized corresponding to the use (periods P2 and P4) and the non-use (periods P1 and P3) of the steam cleaner. The incidences of infection or colonization to *S. capitis* evolved as follows: 1.04 per thousand person-hospital days for P1, 0.55 per thousand person-hospital days for P2, 3.95 per thousand person-hospital days for P3 and 0 per thousand person-hospital days for P4. Incidence was significantly higher during the periods P1 and P3 compared with the periods P2 and P4 (*p* < 0.001). During these different periods, the consumption of vancomycin and linezolid were stable in the NICU.

### Results of laboratory testing and antimicrobial resistance

*S. capitis* isolates were mainly collected from blood (19, 51.3%) and catheters (17, 46.0%). All the strains harboured methicillin and aminoglycoside resistance, while all remained susceptible to fluoroquinolones and linezolid. Resistance to vancomycin was detected for 28 isolates (75.7%). PFGE confirmed that all isolates belonged to *S. capitis* NRCS-A clone*.* No statistical variation in the proportion of microorganisms, CoNS and *S. aureus* isolated during the 4 periods were noticed (Table 2).

**Table 2:** Epidemiological characteristics of preterm infants infected/colonized with *Staphylococcus* spp*.* in Neonatal Intensive Care Unit at Nîmes University Hospital

Characteristics	Period 1^a^	Period 2	Period 3	Period 4	Period 1 & Period 3	Period 2 & Period 4	*p*
	Dec 12-Apr 15	May 15-Nov 16	Dec 16-Feb 17	Mar 17-Dec 17			
Number of microorganisms isolated	1033	598	103	330	1136	928	
Mean of microorganisms isolated per month	36.9 (12–59)	31.3 (19–44)	34.3 (26–36)	33.0 (28–59)	35.6	32.2	ns
Number of *S. aureus* isolated	48	31	5	17	53	54	
Mean of *S. aureus* isolated per month	1.7 (0–5)	1.6 (0–5)	1.7 (0–4)	1.7 (0–4)	1.7	1.7	ns
Number of CoNS^a^ isolated	527	230	44	138	591	368	
Mean of CoNS isolated per month	18.8 (7–41)	12.1 (14–33)	14.7 (13–17)	13.8 (10–24)	16.8	12.95	ns

^a^CoNS, Coagulase negative *Staphylococcus;* ns, not significant corresponding to comparison of incidences between different periods (Period 1, Period 2, Period 3 and Period 4) using Fisher’s exact test

## Discussion

The clone *S. capitis* NRCS-A has emerged as a major cause of HAI in NICUs worldwide [4, 6, 9, 12]. Epidemiological investigations have shown that once present in a NICU, the clone has a large propensity to persist and to reach high prevalence within the setting suggesting the presence of reservoirs inside each NICU [4, 5]. The reasons and mechanisms of its introduction and maintenance specifically in such wards remain unknown.

In the present study, we showed that the disinfection of incubators and heating tables using a steam cleaner system has significantly reduced the presence of *S. capitis* in clinical specimens until its disappearance in period 4 (Table 1). This represents the first evidence of a way that may allow the eradication of this clone from a NICU, as it has already been reported for the fight against vancomycin-resistant *Enterococcus* strains in an Australian NICU [13]. The re-emergence of the *S. capitis* NRCS-A clone during the 3-month period of the none-use of the steam cleaner, due to technical problem with the machine, can be considered as an unexpected temporal “control period” between two periods of use that provides a clear demonstration of the relevance of the system. The succession of NRCS-A-positive also suggests the existence of latent reservoirs close to the settings that could periodically spread to the NICU. The presence of *S. capitis* NRCS-A in the NICU environment has been recently reported inside a NICU in New Zealand, but the effect of disinfection procedures was not explored [9]. Our results suggest that, if the use of a steam cleaner is not able to eradicate the clone in the environment of the NICU, at least it protects the neonates from infection or colonization in their incubators. A larger use of the steam cleaner in the NICU environment deserves to be evaluated to definitively eradicate the NRCS-A clone from the contaminated NICUs.

The steam cleaner may offer special advantages with detergence and disinfection of surfaces in one use. This combination saves time and increases the simplicity of biocleaning processes. Moreover, this system may offer advantages respecting to surface disinfection in health care settings (incubator and heating table), decreasing toxicity and chemical irritation when used around sensitive patient populations, as the neonates, compared to classical chemical products. Furthermore, the efficiency of steam appliances has been approved for the biocleaning of operating blocks, patient rooms, emergency vehicles and sterilization units. Finally, if the cost of one machine is not negligible (≈ $5900), it must be compared to the cost of one infection in the NICU estimated to be approximately $25,000 [14].

Interestingly, in this study, the effect of the steam cleaner seems to be efficient for *S. capitis* epidemiology. This suggests that the environmental contamination is the major factor of colonisation and infection for neonates compared to the other pathogens.

Our study had some limitations: i) it was only observational, but the temporal evolution of incidence in relation to the availability or not of the steam cleaner appeared to be correlated, ii) no environmental sampling was performed to demonstrate effect of steam cleaner on *S. capitis* environmental colonization. However, the recurrence of this microorganism during P3 confirms that the isolate was still present or was permanently reintroduced in the NICU; iii) we did not prove the mattress and/or incubator were the reservoir of *S. capitis*. However, such a study will be possible in the future because a new method of screening for the presence of *S. capitis* NRCS-A has been recently published [15]. To demonstrate that the decrease of *S. capitis* was directly related to the use of the steam cleaner, an interventional cross over study could be proposed, combined with sampling of different surfaces (incubator, mattress, ..) in a NICU.

## Conclusion

In conclusion, our study suggests that the use of the steam cleaner system in complement to continuous infection control interventions was associated with a significant reduction of *S. capitis* NRCS-A in the NICUs. This efficient and safe method should be considered for a widespread and daily use to fight against the Public Health Threatening represented by the NRCS-A clone in NICUs worldwide.
